# Implications of circadian rhythm and stress in addiction vulnerability

**DOI:** 10.12688/f1000research.7608.1

**Published:** 2016-01-13

**Authors:** Darius Becker-Krail, Colleen McClung

**Affiliations:** 1 School of Medicine, Department of Psychiatry, University of Pittsburgh, Pittsburgh, PA, USA

**Keywords:** Addiction, Circadian, Clock, Glucocorticoids, HPA, Reward, Stress, Vulnerability

## Abstract

In the face of chronic stress, some individuals can maintain normal function while others go on to develop mental illness. Addiction, affecting one in every twelve people in America, is a substance use disorder long associated with stressful life events and disruptions in the sleep/wake cycle. The circadian and stress response systems have evolved to afford adaptability to environmental changes and allow for maintenance of functional stability, or homeostasis. This mini-review will discuss how circadian rhythms and stress individually affect drug response, affect each other, and how their interactions may regulate reward-related behavior. In particular, we will focus on the interactions between the circadian clock and the regulation of glucocorticoids by the hypothalamic-pituitary-adrenal (HPA) axis. Determining how these two systems act on dopaminergic reward circuitry may not only reveal the basis for vulnerability to addiction, but may also illuminate potential therapeutic targets for future investigation.

## Introduction

Within any given environment, an organism must learn to anticipate and adapt to external changes/stressors in order to survive. Two systems, the circadian and stress response systems, have evolved to afford adaptability to both recurring and spontaneous environmental changes. Through the active process of allostasis ( McEwen, 1998; Sterling & Eyer, 1988), these systems and many others work to maintain an internal state of stability, or homeostasis, in the presence of these changes. In the face of acute and/or chronic stressors, the ability for an organism to adapt and avoid negative biological consequences is known as resilience. Within the context of psychopathology, resilience can be seen as a person’s ability to maintain physiological and psychobiological homeostasis, in spite of adversity ( Charney, 2004; Feder *et al.*, 2009). While resilient individuals uphold “normal” mental health and homeostatic function when exposed to stress, other individuals may go on to develop neuropsychiatric or behavioral disorders. According to the vulnerability-stress model ( Ingram & Luxton, 2005; Zubin & Spring, 1977), some individuals may be more biologically vulnerable to developing neuropsychiatric disorders when faced with acute and/or chronic stressors ( Pihl & Nantel-Vivier, 2005). To fully understand this phenomenon, current research seeks to understand the effects of stress in the brain (reviewed in McEwen *et al.*, 2015), and elucidate potential structural and/or molecular bases resulting in the vulnerability to develop mental illnesses.

Many neuropsychiatric disorders have long been associated with altered circadian rhythm ( Mills *et al.*, 1977; Van Cauter & Turek, 1986; Wehr *et al.*, 1983) and stress response ( Albus *et al.*, 1982; Amsterdam *et al.*, 1983; Roy *et al.*, 1988); highlighted here, disruptions in both systems are also known to have implications in substance use disorder/addiction and reward-related behaviors ( Briand & Blendy, 2010; Falcón & McClung, 2009; Koob, 2008; Logan *et al.*, 2014). While abnormal circadian and stress response function can independently affect reward-related behavior, understanding the interface of these two systems, during both typical and atypical functioning, may provide greater insight into the complexities of disorder etiology. This mini-review examines the interplay of the circadian and stress response systems, and how this interaction may affect addiction vulnerability.

## Circadian rhythm and the molecular clock

**Figure 1.**
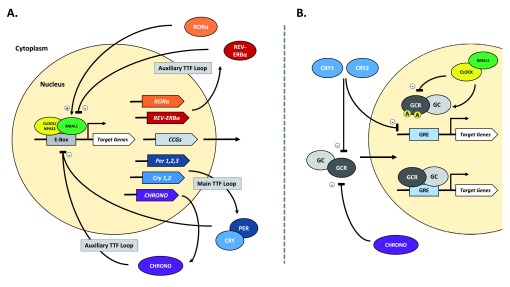
The circadian molecular clock and its interactions with the stress axis. **A**. The mammalian circadian molecular clock consists of multiple transcription and translation feedback (TTF) loops. Central to the TTF loops, the transcription factors CLOCK (or NPAS2) and BMAL1 heterodimerize and bind to the enhancer box (E-Box) sequence to promote transcription of many target and clock controlled genes (CCGs). The main TTF loop is achieved when PERIOD (PER1,2,3) and CRYPTOCHROME (CRY1,2) proteins accumulate, dimerize, undergo phosphorylation, and shuttle back into the nucleus to inhibit both CLOCK/BMAL1 and, as a result, their own transcription. This negative feedback loop cycles every ~24 hours and is crucial for regulation of circadian rhythm. Among its target genes, CLOCK/BMAL1 also regulates the expression of the nuclear receptors RORα and REV-ERBα, both of which can regulate BMAL1 activity via binding at a response element in its promoter. A recently discovered circadian protein, CHRONO, is clock-regulated and can also inhibit CLOCK/BMAL1 activity via interactions at the E-Box. Taken together, these proteins make up auxiliary TTF loops that work to both stabilize and reinforce rhythm. **B**. The circadian molecular clock can interact with the stress axis through regulating activity of both glucocorticoid (GCC) and its receptor (GCR). GC is known to be released under tight circadian regulation, with peak levels in the animal’s active phase. Additionally, several circadian proteins are known to rhythmically regulate GCR-dependent transcription activity. CLOCK/BMAL1 can directly attenuate GCR activity via acetylation (A), thereby reducing its binding ability at the Glucocorticoid Response Element (GRE). Simultaneously, CHRONO and CRY1,2 proteins can repress GCR activity via direct interaction in ligand-fashion. CRY proteins can also regulate GCR-dependent transcription through association at the GRE. (+), promote/activate; (-), repress/inhibit.

Highly conserved across most living organisms, circadian rhythms facilitate the anticipation and adaptation of behavior to daily changes in environmental stimuli. In mammalian organisms, system and cellular level rhythms are maintained by a rhythm-generating nucleus in the hypothalamus, the suprachiasmatic nucleus (SCN). The SCN can be entrained by both photic and non-photic cues called zeitgebers, or “time-keepers”, but can ultimately generate a ~24 hour rhythm independent of these cues. At the molecular level, across all cell types, circadian rhythms are upheld by a “molecular clock” consisting of auto-regulatory transcription-translation feedback loops in the nucleus (see Figure 1A). The key proteins that make up the molecular clock are transcription factors: circadian locomotor output cycles kaput (CLOCK), or neuronal PAS domain protein 2 (NPAS2), and brain and muscle Arnt-like protein 1 (BMAL1). Throughout the day, CLOCK/BMAL1 (or NPAS2/BMAL1) heterodimerize to promote the transcription of *Period* (PER1,2,3), *Cryptochrome* (CRY1,2), and many other clock controlled genes (CCGs). The feedback loop is established when PER and CRY proteins accumulate in the cytoplasm, form hetero- and homodimers, and eventually shuttle back into the nucleus to inhibit their own expression. Additionally, clock regulated RAR-related orphan receptor alpha (RORα) and reverse-ErbA alpha (REV-ERBα) nuclear receptors act in an auxiliary oscillatory feedback loop to regulate expression of *Bmal1,* stabilizing the core feedback loop ( Guillaumond *et al.*, 2005). The circadian molecular clock cycles on a timescale of ~24 hours and regulates the expression of many genes controlling neuronal, metabolic, endocrine, and immune function ( Jin *et al.*, 1999; Lowrey & Takahashi, 2000).

## Circadian genes and reward

Several studies in the past two decades have shown core circadian genes to be important regulators of reward-related behavior in response to common substances of abuse, as reviewed by Parekh *et al.* (2015). One of the first studies investigating this link demonstrated that fruit flies ** with mutations in the *Period*, *Clock*, and/or *Cycle* (similar to *Bmal1* in mammals) genes fail to show behavioral sensitization to cocaine ( Andretic *et al.*, 1999). In mice, mutations in the *mPer1* and *mPer2* genes seem to produce a similar but unique effect on cocaine response; mutation in *mPer2* increased behavioral sensitization to cocaine but mutation in *mPer1* abolished sensitization ( Abarca *et al.*, 2002). Recently, our lab has been studying a similar differential regulation of reward, but with CLOCK and its functional homologue, NPAS2. While CLOCK is known to be expressed almost ubiquitously, NPAS2 is primarily expressed in the liver and forebrain ( Bertolucci *et al.*, 2008; Reick *et al.*, 2001) and can regulate circadian transcription when CLOCK expression is absent or low. With notable implications for reward, NPAS2 has been shown to have a unique expression pattern in the mesolimbic pathway; NPAS2 is highly enriched in the nucleus accumbens (NAc), but has little to no expression in the ventral tegmental area (VTA) ( Garcia *et al.*, 2000).

The observation that NPAS2 is specifically enriched in the NAc motivated our most recent studies investigating the potential for CLOCK and NPAS2 to differentially regulate gene expression and reward-related behavior, following cocaine exposure. Interestingly, in response to chronic cocaine, only NPAS2 is upregulated in the NAc and caudate putamen (normal rhythm abolished) and has increased binding activity at *Period* gene promoters; CLOCK did not show these changes in the NAc or caudate putamen ( Falcón *et al.*, 2013). Knock-down of CLOCK or NPAS2 in these regions also has different effects on the regulation of reward-related behavior following cocaine administration. Mice containing a globally expressed, dominant negative, single-point mutation in the CLOCK protein ( *Clock*∆19) exhibit a mania-like phenotype with increased baseline activity, decreased anxiety- and depression-like behavior, and increased sensitivity to rewarding stimuli; in response to cocaine, *Clock*∆19 mice show increased conditioned place preference (CPP) and increased cocaine self-administration, relative to wild-type mice ( McClung *et al.*, 2005; Ozburn *et al.*, 2012; Roybal *et al.*, 2007). However, using AAV-shRNA to selectively knock-down CLOCK or NPAS2 in the NAc, near entire reduction of CLOCK in the NAc does not recapitulate increased preference seen in *Clock*∆19; while mice with either a mutated form of NPAS2 or a NAc specific knock-down of NPAS2 showed decreased cocaine CPP and self-administration ( Ozburn *et al.*, 2015).

The genes central to the circadian molecular clock may have differential roles in the regulation of reward-related behavior depending on the time of day, brain region, and/or the drug’s effect on the specific protein in a particular region. Given the involvement of these core circadian genes in the regulation of reward and behavioral response, it is likely that disruption to the normal functioning/activity of these circadian proteins can contribute to the vulnerability of developing an addiction.

## HPA axis and reward

When encountering physical and/or psychological stressors, activation of the hypothalamic-pituitary-adrenal (HPA) axis is a fundamental, evolutionarily conserved response allowing the organism to adapt both physiologically and behaviorally. Regulated by the HPA axis, glucocorticoids (cortisol in humans and corticosterone in rodents) are a class of steroid hormones responsible for driving the changes seen in stress response. Upon activation by limbic structures, neurons in the hypothalamic paraventricular nucleus (PVN) release corticotropin-releasing hormone (CRH) at the median eminence into the hypophyseal portal system, connecting the hypothalamus with the anterior pituitary. Once stimulated by CRH, the anterior pituitary increases secretion of adrenocorticotropic hormone (ACTH) into the bloodstream, where it will then travel to the adrenal gland. At the adrenal cortex, ACTH causes an increase in synthesis and release of glucocorticoids into the circulatory system to then act on energy metabolism, protein synthesis, immune function, cardiovascular function, attention/arousal/vigilance, and even memory function. Much like the circadian molecular clock, the HPA axis is regulated via a negative feedback loop in which accumulation of glucocorticoids in the hypothalamus (PVN) and anterior pituitary can block the production of CRH and ACTH. ( de Kloet *et al.*, 2005; Johnson *et al.*, 1992; Uchoa *et al.*, 2014).

While the effects of the HPA axis are transient in response to acute stress, chronic stress and prolonged presence of glucocorticoids can have deleterious outcomes, ranging from neurotoxicity and cell death to impaired metabolic/mitochondrial function and immunosuppression ( de Kloet *et al.*, 2005; Dhabhar & McEwen, 1997; McEwen *et al.*, 1999; Picard *et al.*, 2014). Considering the potential detrimental effects of chronic stress, recent studies have investigated the implications of the HPA axis and glucocorticoids in neuropsychiatric disorder etiology ( Herane Vives *et al.*, 2015; Jacobson, 2014; Sapolsky, 2000; Yehuda *et al.*, 2015). In the context of addiction, animal model studies have long demonstrated a strong association between chronic stress and increased behavioral response to drugs of abuse ( Antelman *et al.*, 1980; Deroche *et al.*, 1995; Herman *et al.*, 1984; MacLennan & Maier, 1983). This increased response can be seen through augmented locomotor effects and increased reward/preference – both of which are associated with elevated glucocorticoid activity ( Deroche *et al.*, 1995; Haile *et al.*, 2001; Lepsch *et al.*, 2005). Further confirming the role of glucocorticoids in this phenomenon, if normal stress-induced increases in corticosterone (in rats) are blocked (via adrenalectomy), the stress-related increase in drug behavioral response is abolished; notably, the response remains intact if normal elevation of corticosterone is artificially maintained ( Deroche *et al.*, 1995; Prasad *et al.*, 1998; Rougé-Pont *et al.*, 1995). Corticosterone has even been shown to be necessary for drug-induced sensitization in general, suggesting its importance in overall behavioral response and drug-seeking behavior, with or without stress as a factor ( de Jong *et al.*, 2009; Przegaliński *et al.*, 2000).

Though it is clear that chronic stress and the biological mediators of stress response may alter one’s sensitivity to the behavioral and/or rewarding effects of drugs of abuse, further investigation into the cellular and molecular bases of this relationship is necessary. Given that the circadian rhythm and stress response systems are both known to be involved in mediating behavioral responses to rewarding-substances, perhaps their interactions may be the key to understanding what drives vulnerability to addiction.

## Circadian regulation of the HPA axis

 Like many other processes in the body, the HPA axis and its hormonal components are under direct circadian regulation by both the SCN and a peripheral clock in the adrenal cortex ( Ishida *et al.*, 2005; Nader *et al.*, 2010; Oster *et al.*, 2006; Son *et al.*, 2008). Glucocorticoids, along with the other hormones in the axis, display a prominent diurnal variation in which peak levels correspond with the organism’s active phase (reviewed in: Kalsbeek *et al.*, 2012; Spiga *et al.*, 2014). Rhythms in glucocorticoids are not only mediated by both direct and indirect projections to the CRH producing PVN neurons at the fore-end of the HPA axis ( Engeland & Arhnhold, 2005; Kalsbeek *et al.*, 2006), but also through modulation of receptors by core circadian proteins (See Figure 1B). Oster *et al.* (2006) demonstrated the importance of a functional adrenal molecular clock in “gating” sensitivity to ACTH (via ACTH receptors) in the adrenal cortex, as means of regulating glucocorticoid synthesis. Moreover, the actual glucocorticoid’s effect across the body can also be regulated through acting on glucocorticoid receptor (GCR) function; multiple studies have demonstrated the ability for CLOCK/BMAL1 to modulate GCRs and their sensitivity via circadian mediated acetylation ( Charmandari *et al.*, 2011; Kino & Chrousos, 2011; Nader *et al.*, 2009). Alongside CLOCK/BMAL1, CRY1,2 have also been shown to repress GCR-dependent transcription activity via association with GCRs and/or at the glucocorticoid responsive element (GRE) ( Lamia *et al.*, 2011). In recent literature, a novel circadian protein named CHRONO, or computationally-highlighted/ChIP-derived repressor of network oscillator, acts as a negative regulator of the molecular clock and may even interact with GCRs to rhythmically repress their function ( Anafi *et al.*, 2014; Goriki *et al.*, 2014). While investigation into CHRONO’s function is still in its early stages, there is potential for this novel protein to play an even greater role in connecting the circadian and stress response systems.

Several circadian-gene mutant mouse studies have further verified the role of core molecular clock proteins in regulating the HPA axis. In *Bmal1* null mutant mice, deficiency in BMAL1 causes a significant reduction in glucocorticoid levels, sensitivity, and altered rhythmicity ( Leliavski *et al.*, 2014); similarly, *Clock* mutant mice lacking functional CLOCK protein also show altered glucocorticoid rhythmicity and decreased total levels ( Oishi *et al.*, 2006; Takita *et al.*, 2013). Along with the core CLOCK/BMAL1 complex, null mutations in the *Cryptochrome* (CRY1,2) or *Period* (PER1) genes also yield altered rhythmicity and, unlike *Clock* and *Bmal1* mutants, increased total glucocorticoid levels ( Dallmann *et al.*, 2006; Destici *et al.*, 2013; Lamia *et al.*, 2011). Even more interesting, cultured BMAL1 and/or PER1,2 deficient mouse embryonic fibroblasts (MEFs) show altered GCR transactivation resulting in hypersensitivity to glucocorticoids ( Han *et al.*, 2014). Taken together, these studies demonstrate the importance of the molecular clock in regulating both the output of the stress axis and its efficacy.

## Stress effects on circadian rhythm 

Occurring simultaneously, the same components of the molecular clock that regulate HPA axis function can also be reciprocally affected by the stress itself. Most notably, chronic stress is known to affect the rhythmic expression of the core circadian genes. A key example can be found through glucocorticoids mediating the expression of *Period* genes. With either acute or chronic stress, levels of *mPer1* and PER1 are elevated in some neural and peripheral tissues ( Al-Safadi *et al.*, 2014; Al-Safadi *et al.*, 2015; Takahashi *et al.*, 2013). PER2 has a similar response to stress ( Segall & Amir, 2010; So *et al.*, 2009) and even loses rhythmic expression in the bed nucleus of the stria terminalis (BNST) and in the amygdala, following adrenalectomy ( Lamont *et al.*, 2005). Additionally, it was later revealed that PER2 actually requires functional GCRs for its rhythmic expression ( Segall *et al.*, 2009). Both *Period* genes are afforded this unique relationship with glucocorticoids due to the presence of GREs in their promotor regions ( So *et al.*, 2009; Yamamoto *et al.*, 2005). Glucocorticoids can target genes that have these GREs, bind, and thus promote their expression. This stress-induced change in ** gene expression, if occurring chronically, can even result in clock entrainment ( Tahara *et al.*, 2015).

Previous work in our lab has shown that mice exposed to chronic social defeat stress show increased anxiety that correlates with decreased *mPer1/2* expression in the NAc ( Spencer *et al.*, 2012). While this may appear to conflict with aforementioned stress effects on *Period* genes, it is likely that region specific changes occur depending on the type of stressor, GCR distribution, and region/network involvement. Illustrating this, we recently showed mice subjected to the unpredictable chronic mild stress (UCMS) paradigm (used to model depression-like behavior) have increased *Per2* rhythm amplitude in the NAc, but decreased rhythm amplitude in the SCN ( Logan *et al.*, 2015). This is likely due to GCR concentration being high in the NAc ( Barik *et al.*, 2010; Der-Avakian *et al.*, 2006) but low/absent in the SCN ( Balsalobre *et al.*, 2000; Pezuk *et al.*, 2012; Rosenfeld *et al.*, 1988). The interaction between stress response and the circadian molecular clock in a region specific manner may perhaps be the basis that allows for manifestation of disorder specific vulnerability.

## Circadian rhythm and stress interactions in dopaminergic transmission

**Figure 2.**
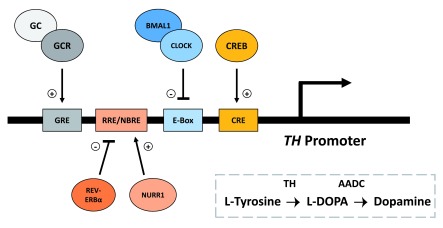
Dynamic circadian and stress interactions at the *TH* promoter. Important for reward-related behavior, tyrosine hydroxylase (TH) is an enzyme involved in the synthesis of dopamine from the amino acid L-Tyrosine. Transcription of *TH* is mediated by the binding of cAMP response element-binding protein (CREB) at its response element (CRE) in the *TH* promoter. Dopamine synthesis is known to be directly regulated by both circadian and stress-related proteins/hormones. At the *TH* promoter, the core circadian CLOCK/BMAL1 complex binds to the enhancer-box (E-Box) sequence in antiphase with CREB:CRE binding, and negatively regulates the transcription of *TH* in a time dependent manner. Additionally, the circadian nuclear receptor REV-ERBα and nuclear receptor-related 1 (NURR1) protein regulate TH expression via competitive binding at the REV-ERB/ROR response element (RRE)/NGF1B-response element (NBRE); while NURR1 promotes the expression of TH, REV-ERBα represses expression. Glucocorticoids (GCs) and its receptor (GCR) can also promote expression of TH by binding at the glucocorticoid response element (GRE) in the *TH* promoter. The above response element spacing is not shown to scale. (+), promote/activate; (-), repress/inhibit.

 As described above, a lot can be understood by just examining how each system not only affects each other, but also how they individually affect behavioral response to drugs of abuse. However, in the framework of addiction and reward, the effects on vulnerability may more likely arise from the two systems’ interactions while simultaneously regulating the same reward-related processes. Both systems have been show to directly regulate/affect the dopaminergic reward circuitry and its functions involved in addiction (reviewed in: Marinelli & Piazza, 2003; Parekh *et al.*, 2014). Implemented in addiction, the mesolimbic pathway is a system of dopaminergic neurons connecting the VTA to the NAc, and is important for mediating reward-related behavior. This pathway’s activity is known to be directly affected by both the circadian system and the stress response system (McClung *et al.*, 2007; Trainor, 2011). In controlling dopamine synthesis, circadian molecular clock proteins and glucocorticoids may be working in opposing fashion to regulate expression of tyrosine hydroxylase (TH), a key enzyme in synthesis of dopamine from tyrosine (see Figure 2).

Under circadian control, recent work in our lab has demonstrated that CLOCK/BMAL1 acts as a time-dependent, negative regulator of *TH* transcription via binding at the *TH* promotor in anti-phase with CREB (Sidor *et al.*, 2015). Opposite of CLOCK/BMAL1, glucocorticoids may positively regulate the expression and activity of TH ( Fossom *et al.*, 1992; Kalinina *et al.*, 2012; Núñez *et al.*, 2009; Tank *et al.*, 1986). While the exact mechanism by which glucocorticoids increase TH expression is still being investigated, it has been shown that the *TH* promotor contains a GRE to which glucocorticoids can bind and promote expression independent of CRE ( Hagerty *et al.*, 2001a; Hagerty *et al.*, 2001b). An additional explanation may arise through glucocorticoid regulation of the circadian nuclear receptor, REV-ERBα. In a recent paper by Chung *et al.* (2014), REV-ERBα has been shown to repress *TH* transcription through competitive binding with nuclear receptor-related 1 protein (NURR1) at the *TH* promotor. Given that glucocorticoids have been shown to control REV-ERBα expression in the liver ( Torra *et al.*, 2000), it may be possible for glucocorticoids to increase TH through a disinhibition type mechanism.

In addition to regulation of TH, the two systems can simultaneously act on dopamine receptor (DR) expression/function ( Biron *et al.*, 1992; Iasevoli *et al.*, 2013; Ikeda *et al.*, 2013; Spencer *et al.*, 2012) and monoamine oxidase-A (MAO-A) expression/function, an enzyme responsible for degradation of dopamine ( Chevillard *et al.*, 1981; Cvijić *et al.*, 1995; Hampp *et al.*, 2008; Soliman *et al.*, 2012). Taken together, these studies demonstrate the interactions of both the circadian system and stress response system in regulating the same aspects of reward related function/behavior. With slight disruption in either of the systems, the negative effects of one can alter the other and result in a self-perpetuating consequence. It is at this level that an understanding of vulnerability to addiction can be more readily obtained.

## Conclusion

Reward-related behavior and sensitivity to drug response have both been shown to be regulated by the circadian and stress response systems. Described above, disruption in either of the systems individually can have pronounced effects on the rewarding effects of substances of abuse. Understanding both the tight circadian regulation of the stress axis and how stress/response can affect the molecular clock, the possibility for their interaction to drive vulnerability to addiction is entirely plausible. Both systems act in parallel regulating many aspects of reward related behavior and function. Slight mutations in either system causing minor functional disruption may be innocuous alone, but given the interface of the two systems, a minor change has the potential to be amplified in a reverberating fashion. The once subtle change may even become deleterious with time. It is in this logic that vulnerability to addiction is rooted; as a biological predisposition that becomes exploited in the face of chronic stressors. As an outlook, future studies can begin to consider one or both systems as potential therapeutic targets to mediate drug response in addiction and combat vulnerability.
