# Supplementary material for: Implications of circadian rhythm and stress in addiction vulnerability
Source: F1000Res. 2016 Jan 13;5:59. [Version 1] doi: 10.12688/f1000research.7608.1 (PMC4743143; doi:10.12688/f1000research.7608.1)
Supplement: Peer review history

# Peer review: Referee response for version 1

**DOI:** 10.5256/f1000research.8194.r11935

**Published:** 2016-02-02

**Authors:** Randy Nelson, Jeremy Borniger

**Affiliations:** 1 Department of Neuroscience, Ohio State University College of Medicine, Columbus, OH, USA

The authors provide an excellent and timely mini-review of the interactions between the circadian and stress response systems, and their actions on dopaminergic reward circuitry underlying addiction and diseases of drug abuse. They highlight the differential expression levels of BMAL1 binding partners CLOCK and NPAS2 throughout the brain, indicating that NPAS2 is specifically enriched in the nucleus accumbens. This sets the stage for the idea that selective manipulation of the circadian clock through NPAS2 in the nucleus accumbens could influence reward-seeking behavior. Additional evidence is discussed regarding the binding of CLOCK/BMAL1 at the tyrosine hydroxylase promoter to repress transcription and therefore dopamine synthesis.

The authors further discuss the fundamental link between the HPA axis and the circadian clock through glucocorticoid-response elements in clock gene and tyrosine hydroxylase promoters, acetylation of the glucocorticoid receptor by CLOCK/BMAL1, and other potential interactions with the newly discovered clock component CHRONO. The review is concise and provides an excellent starting point for anyone interested in the etiology, treatment, or molecular biology of addiction or drug abuse.

It may be beyond the scope of this review, but future discussion of the effects drugs of abuse on the circadian clock may be warranted.

- Is there any evidence for circadian control of opioid reward signaling? It may be worth discussing stress effects on opioid or opioid-receptor expression in the VTA or NAc on a circadian basis. (e.g., Polter *et al,* 2014, Biological Psychiatry Vol 76: 785-793).

We have read this submission. We believe that we have an appropriate level of expertise to confirm that it is of an acceptable scientific standard.

# Peer review: Referee response for version 1

**DOI:** 10.5256/f1000research.8194.r12056

**Published:** 2016-02-01

**Authors:** Harry Pantazopoulos, Syed Bukhari

**Affiliations:** 1 Translational Neuroscience Laboratory, McLean Hospital, Belmont, MA, USA

This is a well written and timely summary of interactions between the circadian system and the stress response system set in the framework of drug addiction/reward. The article provides an accurate summary of the current literature and a helpful introduction of these biological systems to readers who are unfamiliar with the field. The figures are clear, well-described, and aid the reader in understanding the molecular mechanisms described in the text. In addition, the evolution/environmental interaction perspective in the introduction provides an excellent framework for the following discussion of how interactions between the circadian and stress systems can go awry. This mini-review is a useful reference to researchers studying basic neuroscience questions on these systems and how these systems are disrupted in psychiatric disorders. The review highlights the importance of recognizing the potential interactions between these systems in both normal and pathological conditions.

We have read this submission. We believe that we have an appropriate level of expertise to confirm that it is of an acceptable scientific standard.
